# Response to brolucizumab treatment for refractory serous pigment epithelial detachment secondary to polypoidal choroidal vasculopathy

**DOI:** 10.1186/s12886-022-02711-5

**Published:** 2022-12-13

**Authors:** Seung Wan Nam, Zeeyoon Byun, Don-Il Ham, Mingui Kong

**Affiliations:** 1 Department of Ophthalmology, Samsung Medical Center, Sungkyunkwan University School of Medicine, Seoul, Korea; 2 Department of Ophthalmology, Hangil Eye Hospital, 35 Bupyeong-daero, Bupyeong-gu, 21388 Incheon, Republic of Korea; 3 Department of Ophthalmology, College of Medicine, Catholic Kwandong University, Incheon, Korea

**Keywords:** Age-related macular degeneration, Brolucizumab, Optical coherence tomography, Pigment epithelial detachment, Polypoidal choroidal vasculopathy

## Abstract

**Purpose:**

To report the efficacy and safety of brolucizumab in the treatment of refractory serous pigment epithelial detachment (PED) secondary to polypoidal choroidal vasculopathy (PCV).

**Methods:**

Twenty-six eyes of 26 patients were included. Intravitreal brolucizumab 6.0 mg was administered, followed by pro re nata (PRN) retreatment at monthly follow-ups. All patients underwent spectralis domain optical coherence tomography (SD-OCT), fluorescein angiography, and indocyanine green angiography before the first brolucizumab injection. SD-OCT was repeated at follow-up visits. The height and width of the serous PEDs, measured using SD-OCT, were analyzed.

**Results:**

The number of previous anti-VEGF injections was 12.3 ± 15.0. During brolucizumab treatment, anatomical improvement was achieved and maintained in the height and width of the PEDs (*p* < 0.05). However, the visual outcome did not improve significantly (*p* > 0.05). A good response was achieved in 69.2% of eyes at 1 month and at the last visit. Relapse and complete resolution were observed in 27.8 and 23.1% of patients, respectively. The number of brolucizumab injections was 2.00 ± 0.85. Intraocular inflammation, vascular obstruction, and retinal pigment epithelial tears were not observed.

**Conclusion:**

Intravitreal brolucizumab may be an effective and safe treatment option for refractory serous PEDs in patients with PCV.

## Introduction

Polypoidal choroidal vasculopathy (PCV) is a subtype of neovascular age-related macular degeneration (AMD) [1]. It is characterized by a network of branching choroidal vessels with terminal, polyp-like aneurysmal dilations [2]. Optical coherence tomography (OCT) of PCV showed pigment epithelial detachments (PEDs) and exudative changes [1]. Many studies have reported the efficacy and safety of anti-vascular endothelial growth factor (VEGF) treatment in patients with PCV [3, 4].

Retinal PEDs are important predictors of vision loss in patients with AMD [5]. Retinal PEDs are defined as the separation of the retinal pigment epithelium (RPE) from the underlying Bruch membrane layer, and subclassified into serous, drusenoid, fibrovascular, and hemorrhagic PED according to the subretinal material [5]. Retinal PEDs are observed in up to 62.7% of eyes with neovascular AMD (n-AMD) [6], and their presence are often recognized as a progression of n-AMD [7]. Retinal PEDs are poor prognostic factors for anti-VEGF treatment for n-AMD, and are related to suboptimal response, resistance, and tachyphylaxis [8–11], although the presence of a PED has not a significant impact on the functional outcomes (e.g., best-corrected visual acuity [BCVA]) [12].

The responsiveness of retinal PEDs in n-AMD to anti-VEGF treatment has been reported in previous studies [13–17]. Response to anti-VEGF treatment is reportedly dependent on the reflectivity in OCT of retinal PEDs [13–15]. In treatment-resistant PEDs, aflibercept (Eylea; Regeneron, Tarrytown, NY) showed good anatomic outcomes [13, 14]. However, functional outcomes (BCVA) were not significantly improved after several aflibercept injections [13, 14].

The recently developed anti-VEGF agent, brolucizumab (Beovu; Novartis, UK), is a new humanized single-chain variable fragment that shows better tissue penetration and longer durability than previous anti-VEGF agents [18]. Its potential benefits are assumed to be related to its low molecular weight and high molar concentration [18]. Brolucizumab showed a significantly lower rate of disease activity [19], and it also showed significant effects even in refractory n-AMD [20]. Brolucizumab showed good anatomic and functional outcomes in the treatment of n-AMD [21], despite reported safety issues, such as intraocular inflammation (IOI) and retinal vasculitis with or without occlusion,

The purpose of this study was to investigate the efficacy and safety of brolucizumab for serous PEDs refractory to bevacizumab, ranibizumab, and/or aflibercept in patients with PCV.

## Methods

This study was approved by the Institutional Review Board (IRB) of Samsung Medical Center and adhered to the tenets of the Declaration of Helsinki. Given the retrospective design of this study and the use of anonymized data, requirements for informed consent were waived by the IRB of Samsung Medical Center.

We performed a retrospective medical record review of consecutive patients diagnosed with PCV aged over 50 between April 2021 and April 2022 at the Hangil Eye Hospital and Samsung Medical Center. Patients were included if they had serous PEDs secondary to PCV, showed unresponsiveness to other intravitreal anti-VEGF drugs (bevacizumab, ranibizumab, and/or aflibercept), underwent brolucizumab injections and underwent more than 3 months of follow-up after the first brolucizumab injection. If the height of serous PED did not decrease to less than 200 μm in previous anti-VEGF treatments, it was defined as unresponsiveness.

The exclusion criteria were uveitis, severe diabetic retinopathy, severe hypertensive retinopathy, severe epiretinal membrane, retinal detachment, glaucoma, refractive error exceeding ± 6 diopters, history of retinal laser photocoagulation, severe ocular media opacity, and insufficient ocular examinations. Demographic information including age, sex, previous anti-VEGF injections, and comorbidities was obtained for each patient.

### Diagnosis of polypoidal choroidal vasculopathy

PCV was diagnosed according to the CONAN (Consensus on Neovascular Age-related macular degeneration Nomenclature) study group [7]. OCT, fluorescein angiography (FA; Spectralis HRA + OCT), and indocyanine green angiography (ICGA; Spectralis HRA + OCT) were used to confirm the diagnosis of PCV.

### Treatment and follow-up

Intravitreal brolucizumab 6.0 mg was administered, followed by pro re nata (PRN) retreatment at monthly follow-ups. The first follow-up was performed 1 month after the first treatment. PRN retreatment decision was made by the presence of IRF and SRF or increased size of serous PED in OCT images at each follow-up visit. All enrolled patients underwent a comprehensive ophthalmologic evaluation before the first brolucizumab injection. These included measurements of the BCVA, spectral domain optical coherence tomography (SD-OCT; [Spectralis HRA + OCT, Heidelberg Engineering, Heidelberg, Germany]), FA, and ICGA. The BCVA and SD-OCT were repeated to assess responses.

### OCT, FA, and ICGA

The OCT images were obtained using Spectralis HRA + OCT (version 1.7.0.0; Heidelberg Engineering, Heidelberg, Germany), which combines the advantages of confocal scanning laser ophthalmoscopy (cSLO) and SD-OCT to facilitate point-to-point registration analysis. The protocol of SD-OCT consisted of two B-scans centered on the fovea (horizontal and vertical, 12.0 mm, ART 100) and raster scans (30’ x 20’, 6.0 mm, centered at the center of the PEDs, 25 horizontal B-scans, ART 9). Automatic real-time (ART) mode using an eye-tracker system was activated. During the OCT examination, the patients were continuously encouraged to fixate on the internal fixation target. FA and ICGA were performed with simultaneous OCT scans with a maximum density (11 μm distance between the adjacent B-scans) and averaging (100 frames for one B-scan image). Central macular thickness (CMT) was measured at the circular area 1 mm centered to the fovea, acquired from 3D scan protocol, given by the automated software of SD-OCT.

### Analyses of pigment epithelial detachment

Serous PED was identified by a homogeneous hyporeflective subRPE space in the SD-OCT image. If there were more than one serous PED, the highest PED was analyzed. The location of serous PED was classified into subfoveal and extrafoveal lesion. The fovea was defined as the maximum depression and its surrounding area, the diameter of 500 μm.

The height and width of the PEDs were manually measured in a horizontal raster scan image of the highest PED using a caliper tool available on SD-OCT devices. The PED response after treatment was defined as following: “Good response” defined as a decrease in PED height ≥ 50% from baseline; “Partial response” defined as a decrease in PED height < 50% from baseline, and “Poor response” defined as an increase in PED height more than baseline. “Relapse” was defined as a more than two times increase in PED height at any time since showing good response at 1 month. “Complete resolution” was defined as a completely flattened PED without empty space between the RPE and Bruch’s membrane at any time.

All measurements were assessed by two independent retinal specialists (S.W.N. and M.K.). Inter- and intragrader agreement on each measurement was regularly assessed, and consensus training was initiated when κ values were below 0.6. All uncertain diagnoses were adjudicated by a senior interpreter (D-I.H.).

### Statistical analyses

Quantitative variables are presented as mean ± standard deviation. Frequencies were compared between groups using the chi-square or Fisher’s exact test. Analyses of continuous variable changes were performed using independent t-tests. Statistical significance was defined as a *p*-value of < 0.05. All statistical analyses were performed using SPSS software (version 20.0; SPSS, Chicago, IL, USA).

## Result

Twenty-six eyes of 26 patients with refractory serous PEDs in PCV were enrolled in this study. The mean age of the population was 69.0 ± 7.6 years. The mean baseline BCVA was 0.31 ± 0.28 logMAR, mean PED height at baseline was 485.6 ± 179.2 μm, and mean PED width at baseline was 2672.8 ± 1129.6 μm. All eyes had previously received anti-VEGF injections. One eye was treated with photodynamic therapy. Table 1 shows the baseline characteristics of the enrolled patients.

**Table 1:** Baseline demographics of serous pigment epithelial detachment (PED) secondary to polypoidal choroidal vasculopathy (PCV)

	Serous PEDs secondary to PCV (*n* = 26)
Age (years)	69.0 ± 7.6 [52.0–85.0]
Male (%)	15/26 (57.7%)
Lens status (phakia)	17/26 (65.4%)
Mean BCVA (logMAR)	0.31 ± 0.28 [0.05–1.30]
Baseline BCVA (logMAR), n (%)	
< 0.40 (20/40)	20 (76.9%)
0.40 (20/40) to 1.0 (20/200)	5 (19.2%)
> 1.0 (20/200)	1 (3.8%)
CMT at baseline (µm)	515.5 ± 235.7 [230–1085]
PED height at baseline (µm)	485.6 ± 179.2 [231–953]
PED width at baseline (µm)	2672.8 ± 1129.6 [1154–5169]
PED location, n (%)^a^	
Subfoveal	19 (73.1%)
Extrafoveal	7 (26.9%)
The number of previous anti-VEGF injections	12.3 ± 15.0 [1.0–71.0]
The duration of previous anti-VEGF injections (months)	22.57 ± 10.46
The interval between the last previous anti-VEGF injection and the first brolucizumab injection (months)	2.00 ± 0.00

Data are total no. (%) or mean ± standard deviation, unless otherwise indicated

*PED* Pigment epithelial detachment, *PCV* Polypoidal choroidal vasculopathy, *BCVA* Best corrected visual acuity, *logMAR* Logarithm of the minimum angle of resolution, *CMT* Central macular thickness, *VEGF* Vascular endothelial growth factor

^a^ If any part of PED involved subfoveal area, the location of PED was defined as subfoveal

### Response to brolucizumab treatment

**Fig. 1:**
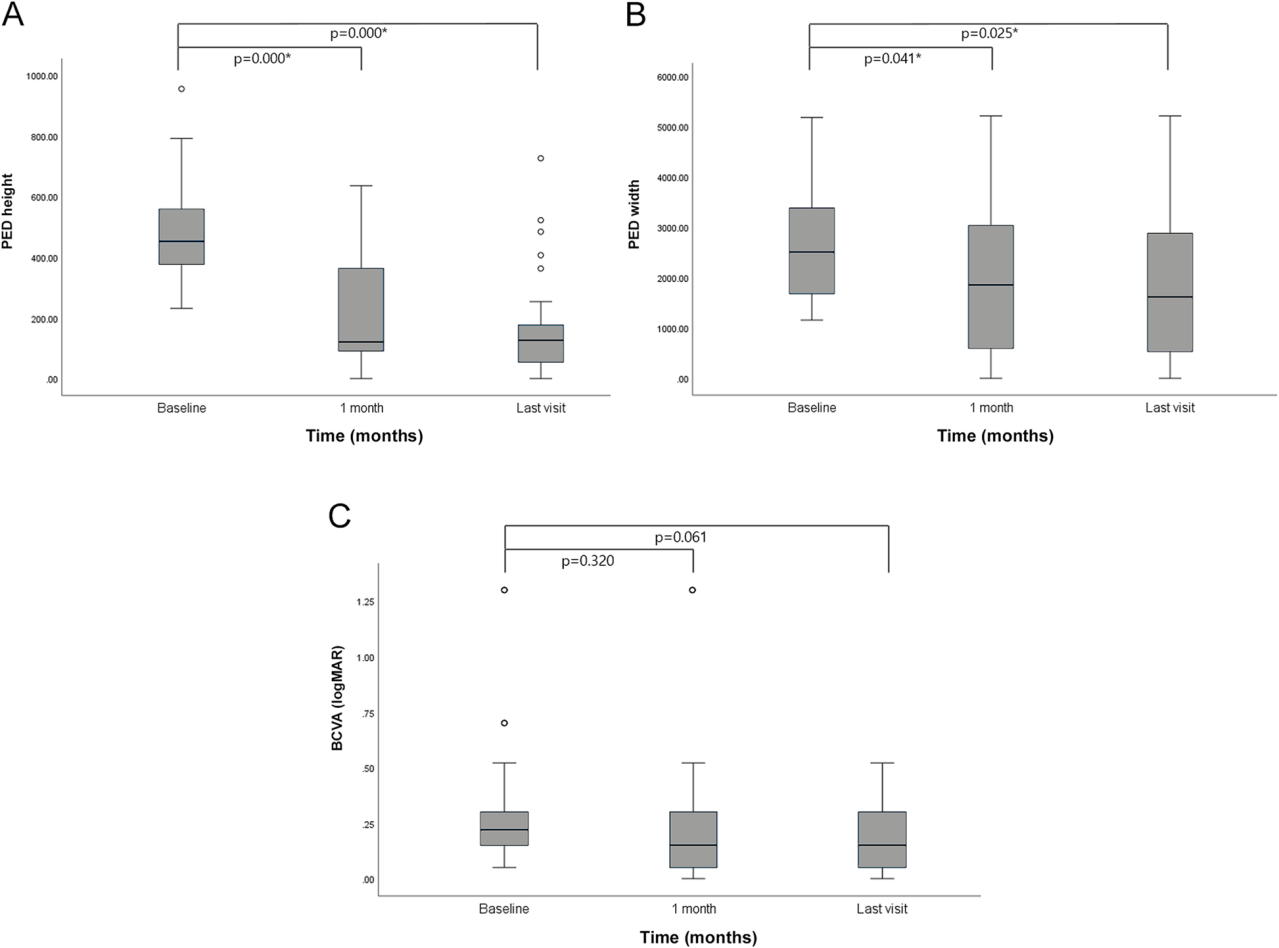
Anatomical and functional outcomes of serous pigment epithelial detachment (PED) in polypoidal choroidal vasculopathy (PCV) at baseline and follow-up visits with brolucizumab injections. Anatomical improvement was achieved and maintained in the height and width of pigment epithelial detachment (*p* < 0.05). However, no functional improvement was observed (*P* > 0.05)

The PED height decreased significantly during the study period. The mean PED height decreased from 485.6 ± 179.2 μm at baseline to 190.7 ± 172.2 μm at 1 month (*p* = 0.000), and 172.4 ± 183.7 μm (*p* = 0.000) at the last visit. The PED width decreased significantly during the study period. The mean PED width decreased from 2672.8 ± 1129.6 μm at baseline to 1907.4 ± 1480.9 μm at 1 month (*p* = 0.041), and 1821.0 ± 1497.8 μm at the last visit (*p* = 0.025). Whereas the BCVA (logMAR) showed a continuous reduction during the follow-up period, it did not improve significantly (*p* > 0.05). The BCVA (logMAR) was changed from 0.31 ± 0.28 logMAR at baseline to 0.24 ± 0.27 logMAR at 1 month (*p* = 0.320) and 0.19 ± 0.16 logMAR at the last visit (*p* = 0.061) (Fig. 1).

The percentage of patients with a good PED response was 69.2% (18 eyes) at 1 month and at the last visit. The percentage of partial PED response was 23.1% (6 eyes) at 1 month and at the last visit. The percentage of patients with a poor PED response was 7.7% (2 eyes) at 1 month and at the last visit. The patients who showed a good response at 1 month mostly presented with a good response at the last visit (83.3% (15/18)). The patients who showed a partial response at 1 month presented with a 50.0% (3/6) of good responses at the last visit. All patients with a poor response at 1 month presented with a poor response at the last visit (100.0% (2/2)).

Relapse was observed in 27.8% (5/18) of the patients who showed a good response at 1 month. Among them, 60.0% (3/5) showed a good response at the last visit, and 40.0% (2/5) showed a partial response at the last visit. Complete resolution was achieved in 23.1% (6/26) of the patients. The mean time and number of brolucizumab injections to achieve complete resolution were 1.50 ± 0.84 [1.00–3.00] months and 1.17 ± 0.41 [1.00–2.00] injections, respectively. The BCVA change between complete resolution and baseline was − 0.18 ± 0.19 logMAR. Visual improvement was observed in all patients in a complete response group without statistical significance (*p* = 0.107).

The development of RPE tears, intraocular inflammation (IOI), retinal vasculitis, and vascular obstruction was not observed. CMT decreased from 515.5 ± 235.7 μm at baseline to 285.9 ± 103.1 μm at the last visit (*p* = 0.000).

**Fig. 2:**
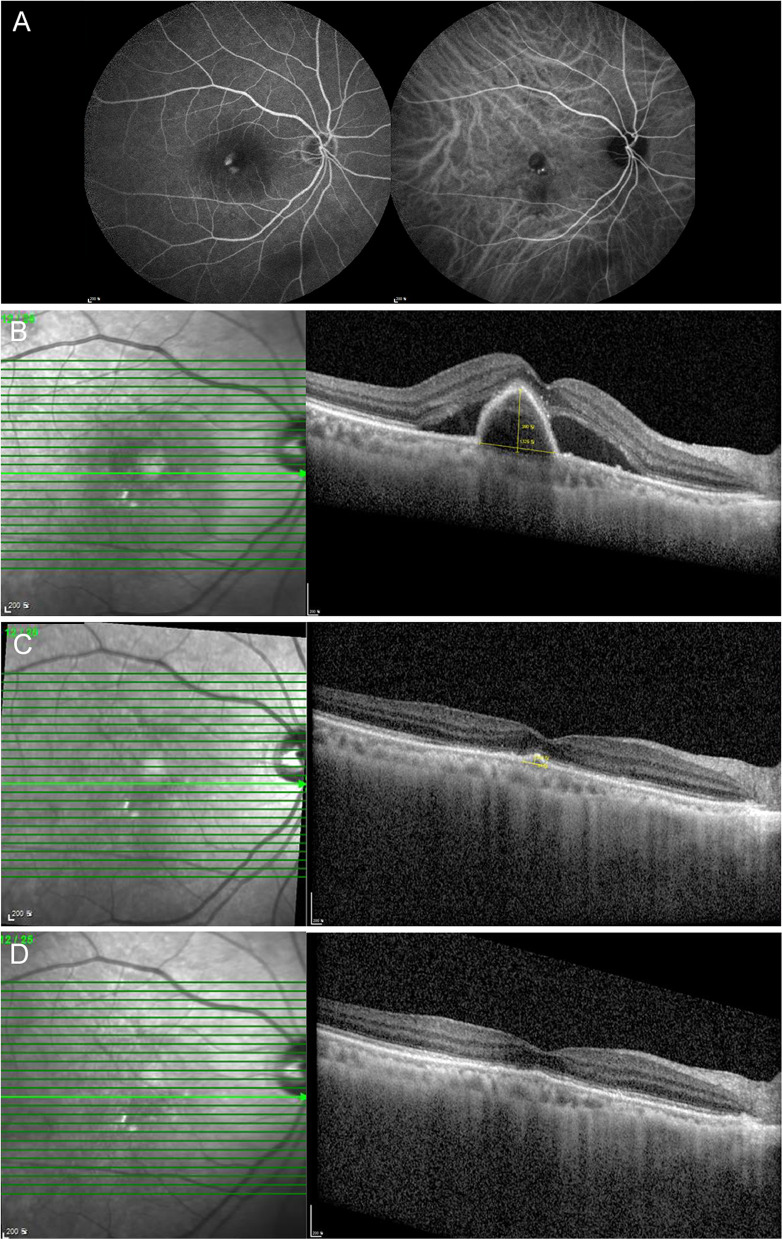
A representative case of serous pigment epithelial detachments (PEDs) showing a good response to brolucizumab. A 72-year-old woman presented with serous PED with polypoidal choroidal vasculopathy (PCV). Despite three previous aflibercept injections, the serous PED was not resolved. Two months after the last aflibercept injection, the treatment was changed to brolucizumab. **A** Fundus fluorescein angiography and indocyanine green angiography shows polypoidal macular neovascularization. **B** Optical coherence tomography (OCT) shows a large serous PED with subretinal fluid (SRF) on the day of the first injection of brolucizumab. **C** A month after the first injection of brolucizumab, the serous PED and SRF improved markedly but persisted. **D** At the last visit, 3 months after the first brolucizumab injection, the serous PED completely disappeared

**Fig. 3:**
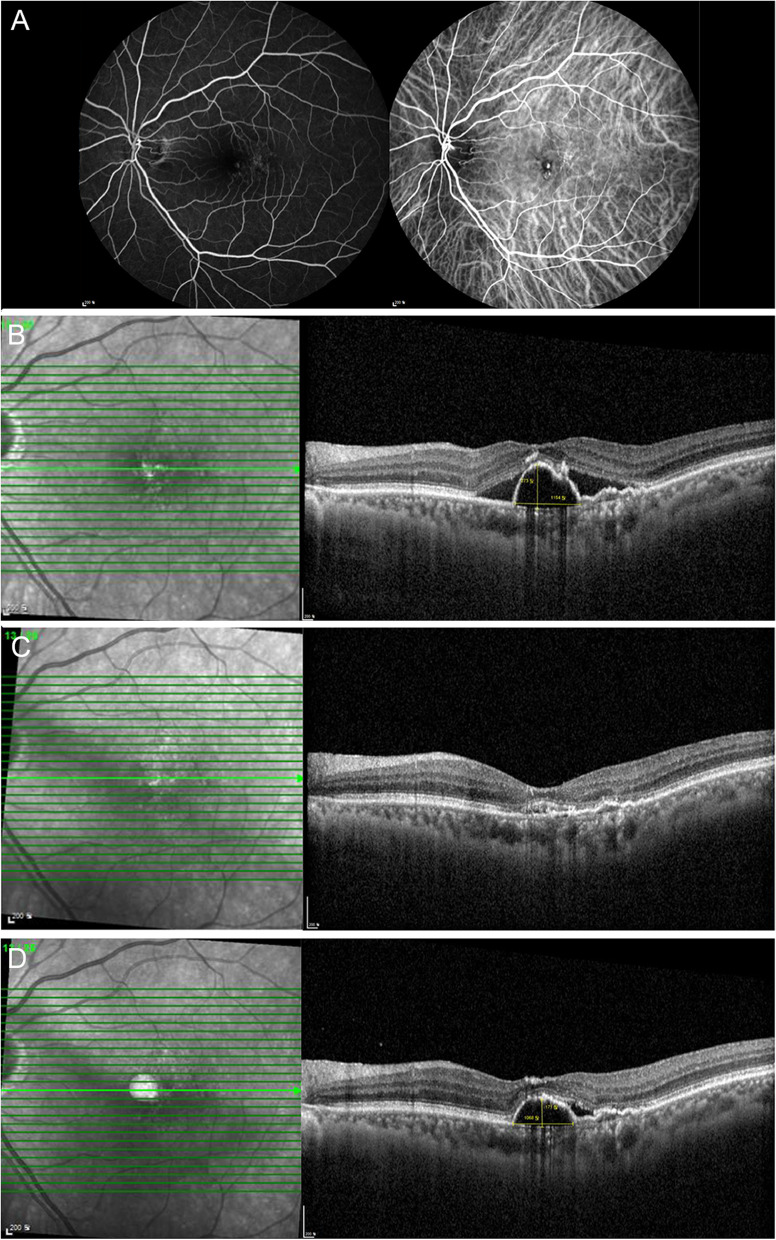
A representative case of serous pigment epithelial detachments (PEDs) showing relapse after a good response to brolucizumab. A 65-year-old man presented with serous PED and polypoidal choroidal vasculopathy (PCV). Despite 23 aflibercept and 8 bevacizumab injections, the serous PED was not resolved. Two months after the last aflibercept injection, the treatment was changed to brolucizumab. **A** Fundus fluorescein angiography and indocyanine green angiography shows polypoidal macular neovascularization. **B** Optical coherence tomography (OCT) shows a large serous PED with subretinal fluid (SRF) on the day of the first injection of brolucizumab. **C** A month after the first injection of brolucizumab, serous PED and SRF markedly improved. **D** At the last visit, 3 months after the first brolucizumab injection, serous PED and SRF redeveloped

**Fig. 4:**
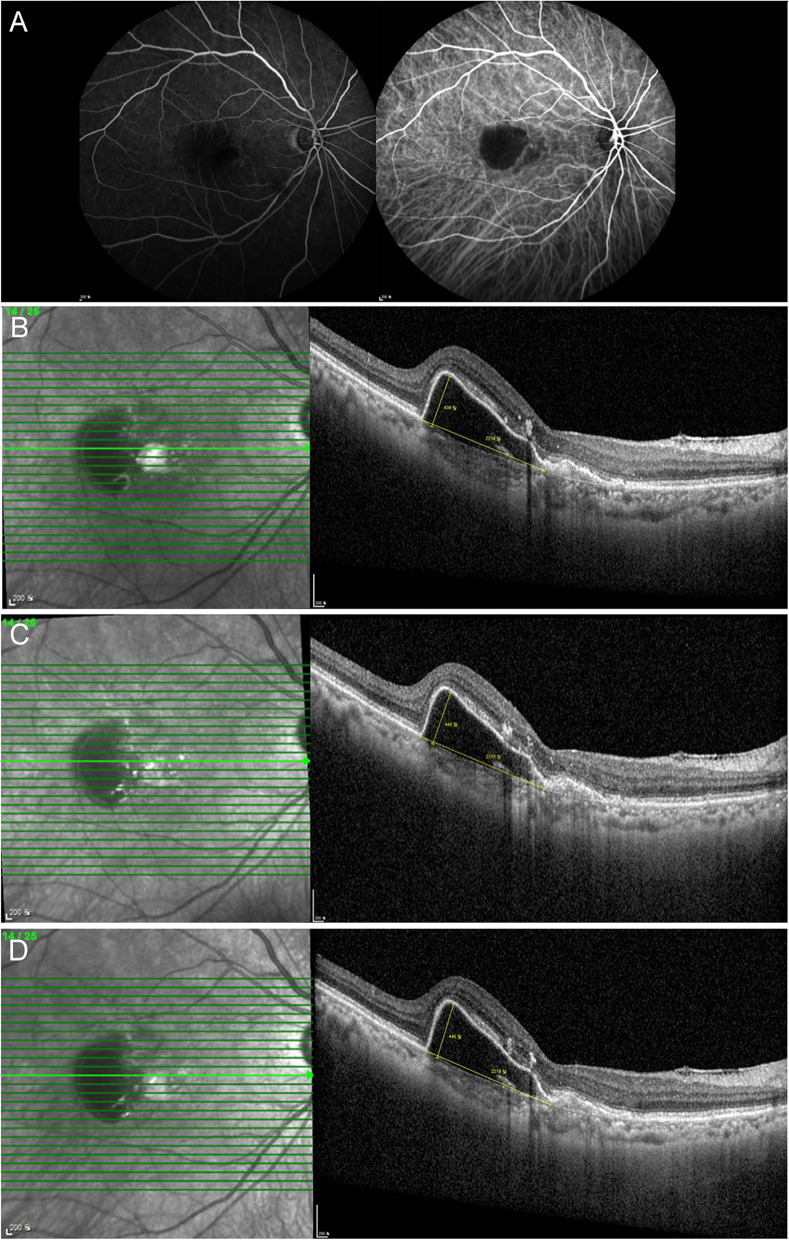
A representative case of serous pigment epithelial detachments (PEDs) showing a poor response to brolucizumab. A 70-year-old woman with serous PED and polypoidal choroidal vasculopathy (PCV). Despite 19 aflibercept and 9 bevacizumab injections, the serous PED was not resolved. Two months after the last aflibercept injection, the treatment was changed to brolucizumab. **A** Fundus fluorescein angiography shows blocked fluorescence due to serous PED, and indocyanine green angiography shows tiny dot-like polyps. **B** Optical coherence tomography (OCT) reveals a large serous PED on the day of the first injection of brolucizumab. **C** A month after the first injection of brolucizumab, serous PED did not respond. **D** At the last visit, 2 months after the third brolucizumab injection, the serous PED did not respond

Overall, the mean number of brolucizumab injections was 2.00 ± 0.85. The mean follow-up duration was 4.27 ± 2.42 months. Representative cases are presented below (Figs. 2, 3 and 4). Participants’ responses are summarized in Table 2.

**Table 2:** Response to brolucizumab treatment of serous pigment epithelial detachments (PEDs) in polypoidal choroidal vasculopathy (PCV)

	Serous PEDs secondary to PCV (n = 26)
PED height change from baseline at last visit	-18.3 ± 126.0 [-325.0–171.0]
PED width change from baseline at last visit	-851.8 ± 1106.1 [-4591.0–237.0]
BCVA (logMAR) change from baseline at last visit	-0.12 ± 0.17 [-0.78–0.15]
PED response at 1month, n (%)^a^	
Good response	18 (69.2%)
Partial response	6 (23.1%)
Poor response	2 (7.7%)
PED response at last visit, n (%)^a^	
Good response	18 (69.2%)
Partial response	6 (23.1%)
Poor response	2 (7.7%)
Relapse^b^	5/18 (27.8%)
Complete resolution^c^	6/26 (23.1%)
Development of RPE tear, n (%)	0 (0.0%)
Development of IOI, retinal vasculitis, and vascular obstruction, n (%)	0 (0.0%)
Number of brolucizumab injections	2.00 ± 0.85 [1–3]
Follow-up duration (months)	4.27 ± 2.42 [3.00–9.90]

Data are total no. (%) or mean ± standard deviation, unless otherwise indicated

*PED* Pigment epithelial detachment, *PCV* Polypoidal choroidal vasculopathy, *BCVA* Best corrected visual acuity, *logMAR* Logarithm of the minimum angle of resolution, *VEGF* Vascular endothelial growth factor, *RPE* Retinal pigment epithelium, *IOI* Intraocular inflammation

^a^ Response was defined as the following criteria; Good response: A decrease in PED height ≥ 50% from baseline. Partial response: A decrease in PED height < 50% from baseline. Poor response: An increase in PED height more than baseline

^b^ Relapse defined as a more than double the increase in PED height since showing good response at any time

^c^ Complete resolution defined as a completely flattened PED without empty space between the RPE and Bruch membrane at any time

## Discussion

In this study, refractory serous PED secondary to PCV showed a good overall response after brolucizumab treatment, without serious adverse events. Early response to PED height is important for predicting future prognosis. The relapse of PED was managed with additional brolucizumab treatment, and these patients showed good final results. Complete resolution of PED was achieved in some cases and was related to visual improvement.

Neovascular AMD may show intraretinal fluid (IRF), subretinal fluid (SRF), and/or PED [22]. PED can occur in up to 62.7% of eyes with n-AMD [6]. The treatment for PED is unsatisfactory, although multiple anti-VEGF agents are available [23]. The previous study reported that the PED type, n-AMD subtype, baseline PED height, and anti-VEGF drug type were associated with the probability of PED resolution after anti-VEGF treatment [24]. Serous PED, PCV, low PED height, and aflibercept treatment showed higher probability of PED resolution [24]. A previous study suggested that these differences were caused by the pathogenesis of PED and binding affinity of anti-VEGF [24]. In this study, we enrolled only patients with serous PED secondary to PCV refractory to previous anti-VEGF treatment, and treated patients only with brolucizumab, a new anti-VEGF agent, which showed favorable anatomic and functional outcomes in n-AMD [18]. Owing to its high retinal permeability, brolucizumab may have advantages over other anti-VEGF agents in PED treatment [18]. In the previous study of brolucizumab, early morphofunctional changes occurred after brolucizumab treatment in patients with n-AMD previously treated with other anti-VEGF [25].

PED treatment should be considered in cases with increasing PED size and associated vision loss [26]. In this study, despite numerous treatments, patients experienced vision loss due to large serous PED before brolucizumab treatment. Therefore, we changed the anti-VEGF antibody to brolucizumab. Previous studies have reported that the treatment of PED did not always correlate with good visual outcomes [24, 26], and in this study, we obtained similar results. During brolucizumab treatment, the PED height and width decreased at 1 month (*p* < 0.05) and were maintained at the last visit (*p* < 0.05). However, the BCVA did not improve significantly at 1 month (*p* > 0.05) or at the last visit (*p* > 0.05). Although the BCVA improvement was not statistically significant, visual improvement was observed in this study. This seems to be due to the lack of visual improvement in general, although some patients showed improved vision. Longer follow-up durations and large-scale studies are necessary to reveal the relationship between functional outcomes (e.g., vision) and brolucizumab treatment.

In this study, brolucizumab showed significant response rates to the PED height. The response at 1 month after brolucizumab treatment is important to predict the prognosis. A previous study of exudative AMD reported that the PED height was significantly reduced within 1 month after the first brolucizumab injection [27]. It is speculated that one month could be enough to see the responsiveness of brolucizumab. Therefore, long-term and large-scale studies are necessary in future.

Relapse was observed in 27.8% (5/18) of patients in this study. Among them, 60.0% (3/5) showed a good response at the last visit, and 40.0% (2/5) showed a partial response at the last visit. These results indicate that PED relapse can be properly managed with repeated brolucizumab injections. Moreover, patients with refractory PED secondary to PCV should be followed-up with thorough examination after brolucizumab injections because of the possibility of relapse.

Complete resolution was observed in 23.1% (6/26) of patients in this study. These results are slightly higher than those of a previous study with aflibercept and ranibizumab (19.3%) [24]. It is difficult to compare directly because of different patient selection criteria, sample size, and follow-up duration. However, as we selected patients who did not respond to other anti-VEGF agents, brolucizumab may have had a stronger effect on PED than other anti-VEGF agents. Lesser time (1.50 ± 0.84 [1.00–3.00] months) and fewer number of brolucizumab injections (1.17 ± 0.41 [1.00–2.00]) were required to obtain complete resolution in this study. Stepwise improvement was also observed in two cases. A higher complete resolution rate could be achieved with more injections of brolucizumab if a longer follow-up duration had been made. The response appeared quickly in most cases, although it improved after several injections in some other cases. Therefore, it is recommended to try additional treatment if there is a response of PED to brolucizumab treatment. In this study, all cases of complete resolution showed visual improvement, even though there was no improvement of vision in the entire group (*p* > 0.05). Cho et al. also reported that complete resolution of PED with anti-VEGF treatment was related to visual improvement [24].

RPE tear is an important issue during anti-VEGF treatment owing to its association with poor visual prognosis [24]. RPE tears could occur in 15–20% of eyes with PED after anti-VEGF treatment, especially in the eyes with PED height ≥ 600 μm [26]. RPE tears occur more frequently with aflibercept than with ranibizumab and high-dose anti-VEGF treatment [24, 28]. Owing to the high effectiveness of brolucizumab, RPE tear is a concern for clinicians. The HAWK and HARRIER trial reported RPE tears in 0.5% of n-AMD treated with brolucizumab [18]; however, in this study, RPE tears were not observed (0.0%). Further studies are necessary owing to the small sample size and short follow-up duration.

Intraocular inflammation and retinal vasculitis with or without occlusion are the major adverse events in brolucizumab treatment [18]. HAWK and HARRIER trial reported IOI in 2.2% [18]. In this study, IOI, retinal vasculitis, and vascular obstruction were not reported (0.0%). This may be because the study population and the number of brolucizumab injections was small. Further large-scale studies are necessary to identify the safety risks of brolucizumab treatment in PEDs.

To our knowledge, this study is the first to investigate brolucizumab treatment of serous PEDs in PCV; however, it had several limitations. First, as it was an observational study, not all patients received the same treatment or had the same follow-up schedule. The PED responses may differ depending on the number and interval of brolucizumab injections. Second, we could not measure choroidal thickness on SD-OCT, which may be related to the responsiveness of serous PED in PCV due to the shadow effect of large serous PED. Third, owing to the small sample size, the statistical power was insufficient to reveal the baseline characteristics of the poor response group to brolucizumab treatment. Fourth, the follow-up period was short (4.27 ± 2.42 months).

## Conclusion

In conclusion, serous PEDs secondary to PCV showed significant anatomical improvements after brolucizumab injections. Functional improvement was not clear, but was observed in cases with complete resolution of PED. Response at 1 month after brolucizumab treatment is important to predict future prognosis, and relapse can be treated with additional brolucizumab treatment. RPE tears, IOI, and retinal vasculitis/obstructions were not observed. Further studies are necessary to better understand the mechanisms and responses of serous PED secondary to PCV to brolucizumab treatment.

## Data Availability

The datasets generated and/or analysed during the current study are not publicly available due to privacy or ethical restrictions but are available from the corresponding author on reasonable request.
